# Comparison of predictive modeling approaches for 30-day all-cause non-elective readmission risk

**DOI:** 10.1186/s12874-016-0128-0

**Published:** 2016-02-27

**Authors:** Liping Tong, Cole Erdmann, Marina Daldalian, Jing Li, Tina Esposito

**Affiliations:** Advocate Health Care, 3075 Highland Parkway, Downers Grove, IL 60515 USA; Cerner Corporation, World Headquarters, 2800 Rockcreek Parkway, North Kansas City, MO 64117 USA

**Keywords:** Predictive Models, Readmission Risk, STEPWISE, LASSO, Ada Boost

## Abstract

**Background:**

This paper explores the importance of electronic medical records (EMR) for predicting 30-day all-cause non-elective readmission risk of patients and presents a comparison of prediction performance of commonly used methods.

**Methods:**

The data are extracted from eight Advocate Health Care hospitals. Index admissions are excluded from the cohort if they are observation, inpatient admissions for psychiatry, skilled nursing, hospice, rehabilitation, maternal and newborn visits, or if the patient expires during the index admission. Data are randomly and repeatedly divided into fitting and validating sets for cross validations. Approaches including LACE, STEPWISE logistic, LASSO logistic, and AdaBoost, are compared with sample sizes varying from 2,500 to 80,000.

**Results:**

Our results confirm that LACE has moderate discrimination power with the area under receiver operating characteristic curve (AUC) around 0.65-0.66, which can be improved to 0.73-0.74 when additional variables from EMR are considered. These variables include Inpatient in the last six months, Number of emergency room visits or inpatients in the last year, Braden score, Polypharmacy, Employment status, Discharge disposition, Albumin level, and medical condition variables such as Leukemia, Malignancy, Renal failure with hemodialysis, History of alcohol substance abuse, Dementia and Trauma. When sample size is small (≤5000), LASSO is the best; when sample size is large (≥20,000), the predictive performance is similar. The STEPWISE method has a slightly lower AUC (0.734) comparing to LASSO (0.737) and AdaBoost (0.737). More than one half of the selected predictors can be false positives when using a single method and a single division of fitting/validating data.

**Conclusions:**

True predictors can be identified by repeatedly dividing data into fitting/validating subsets and referring the final model based on summarizing results. LASSO is a better alternative to the STEPWISE logistic regression, especially when sample size is not large. The evidence for adequate sample size can be explored by fitting models on gradually reduced samples. Our model comparison strategy is not only good for 30-day all-cause non-elective readmission risk predictions, but also applicable to other types of predictive models in clinical studies.

## Background

According to the report from Healthcare Cost and Utilization Project (HCUP), about $41.3 billion in hospital costs were associated with approximately 3.3 million adult all-cause 30-day hospital readmissions in the United States [18]. Hospital were strongly motivated to reduce the readmission rate by the Centers for Medicare and Medicaid Services (CMS) payment methods that reward hospitals with less readmissions while punish those with excessive readmissions in conditions such as acute myocardial infarction (AMI), heart failure (HF), and pneumonia (PN). This list has been expanded to include chronic obstructive pulmonary disease (COPD) and elective primary total hip and/or total knee arthroplasty (THA/TKA) in 2015 and coronary artery bypass graft (CABG) surgery in 2017.

Interventions at and after discharges, such as education on readmission prevention, discharge follow-up appointments, transitional nurse visit, in-time completion of discharge forms and various care management programs were designed to reduce readmission risk. To make interventions most effective, it is very important for hospitals to allocate limited resources to patients with the most need. Therefore, accurate identification of patients at high risk of readmissions is the basis of any successful intervention program.

One method of identifying a patient’s risk of readmission is through the use of predictive models. To evaluate and compare the accuracy of these risk prediction models, two major components must be considered: discrimination and calibration [7]. Discrimination measures how well the model can separate the high and low risk groups, which is often measured by the area under the receiver operating characteristic (ROC) curve, denoted by AUC. Calibration measures how well the predicted probabilities agree with observed risk, which can be measured by statistics such as Hosmer-Lemeshow statistic [20] or Brier score [3]. Although criticized by Cook [7] for its overuse, AUC is still of major interest in readmission risk prediction literature because the main goal is usually to classify patients into high or low readmission risk groups and it is less important to have an accurate match of predicted and observed risks for a random subgroup.

Current readmission risk prediction models have mostly focused on logistic models because the dependent variable of 30-day readmission is usually binary indicating yes or no. The predictors are either pre-determined by experience [8, 26] or selected using a stepwise variable selection strategy from a limited pool of variables [1, 23, 24]. The experience based models usually focus more on the understanding, instead of predicting, of readmission risk and therefore have low prediction accuracy [22, 26]. The stepwise strategies can be problematic and fail to identify true predictors when the sample size is not large enough [9, 16].

In fact, variable selection has been a traditional but very important problem in the field of statistics. The theory and procedures of variable selection in fitting and predicting models have been extensively discussed and evaluated by statisticians [4, 15, 17]. However, in the medical literature, many researchers simply use whatever is available for variable selection, which usually ends up with forward, backward or backward-forward strategies that work well for simple models with a limited number of independent variables. But with the emergence of EMR and a considerable increase in the amount of available data, the stepwise strategy may not be an optimal choice in referring models. Instead, shrinkage methods such as LASSO [27] or machine learning strategies such as Ada boosting [10, 25] can be better alternatives.

Note that throughout the paper, we use the lower case word “stepwise” to indicate forward, backward or forward-backward selections, and the upper case word “STEPWISE” to represent forward-backward selection only. In the machine learning methods, we choose LASSO over the elastic net criterion because in medical practice we would rather to shrink down the number of predictors whenever possible to make it easier when accommodating various types of stored medical information. In addition, some other competing computational methods, such as Random Forest, can also perform well in predictive modelling with collinearity and variables of mixed-type [2]. However, the black box prediction is less preferable in medical practice due to its limitation on explanations of predictors, while the AdaBoost method has been shown to have an approximate parametric format that is similar to logistic models with easy interpretations [13].

The idea of LASSO is to penalize the absolute size of the regression coefficient so that some of the coefficient estimates can shrink towards zero. Specifically, consider the typical set up for linear regression. Let *Y* be the dependent variable, *X* the independent variables (predictors), *n* the number of subjects (sample size), and *p* the number of predictors. The linear regression model assumes *E*(*Y*|*X* = *x*) = *β*_0_ + *x*^*T*^*β* and the estimated parameter $\widehat{\beta}$ (a vector with length *p*) is the one that minimizes the sum of deviation squares ∑_*i* = 1_^*n*^(*y*_*i*_ − *β*_0_ − *x*_*i*_^*T*^*β*)^2^ over the space of *β*. However, the LASSO penalty is to minimize ∑_*i* = 1_^*n*^(*y*_*i*_ − *β*_0_ − *x*_*i*_^*T*^*β*)^2^/(2*n*) + *λ*∑_*j* = 1_^*p*^|*β*_*j*_|, where *λ* is a tuning parameter that can be determined using cross validation. This is convenient for automatic variable selection procedure.

The AdaBoost is a computational algorithm to combine weak classifiers into a powerful one [11]. The weak classifiers are defined as rules or models that classify the data with errors better than random guessing. When adding a new classifier, the AdaBoost focuses on difficult data points that have been most misclassified by the previous weak classifier. Finally, the AdaBoost combines these weak classifiers into a single one using an optimally weighted majority vote. The AdaBoost is fast, simple and easy to use. In addition, it carries out variable selection during the fitting process without relying on heuristic techniques [19].

To make our results comparable with other literature, we also include the LACE model in the comparison [27]. The LACE score was originally designed in 2010 for risk of death or unplanned readmission within 30 days after discharge. It became popular soon due to its simplicity and reasonable discrimination power. The four predictors considered in LACE are (1) Length of stay (L), (2) Acute admission (A), (3) Charlson comorbidity index (C), and (4) Emergency department visits in the past 6 months (E). Points are assigned according to values of these four variables. The final LACE score is calculated by summing the points of all four predictors. LACE scores vary from 0 to 19 where the higher the score, the higher the risk.

In this paper, we consider the hospital 30-day all-cause non-elective readmission as the dependent variable. The independent variables include all patient administrative and medical data, which result in more than three hundred potential predictors. We use the LACE model as the baseline to explore the necessity of using EMR to improve the prediction of readmission risk. We then compare the predictive performance for models based on STEPWISE, LASSO and AdaBoost for samples with various sizes. This study has been determined by the Advocate Health Care IRB office to be exempt from IRB review and approval because no PHI was included and there were no protocol-specific patient interventions.

## Methods

The data are extracted from eight Advocate Health Care hospitals located in the Chicago metropolitan area: Condell Medical Center, Good Shepherd Hospital, Lutheran General Hospital, Illinois Masonic Medical Center, Good Samaritan Hospital, Christ Medical Center, Trinity Hospital and South Suburban Hospital. The full data set include 109,421 adult inpatients (162,466 index admissions) discharged from March 1^st^, 2011 to July 31^st^, 2012. Note that the number of index admissions is more than the number of patients because a patient can have multiple index admissions in the study period. Index admissions are excluded from the cohort if they are observation, inpatient admissions for psychiatry, skilled nursing, hospice, rehabilitation, maternal and newborn visits, or if the patient expires during the index admission. The independent variables include patients’ administration variables, current and history of conditions, procedure and medication variables, and lab test results. The total number of variables is 273, which expands to 325 after k-class categorical variables are converted to (k–1) dummy variables.

An example for the definition of index admissions and readmissions is displayed in Table 1. For example, encounter 2 is not a readmission because it is elective. Encounter 4 is not a readmission because its previous encounter is beyond 30 days. Encounter 5 is a readmission following encounter 4 but not an index admission because its discharge date is not within our study period. There are correlations between multiple index admissions of the same patient. One way to deal with this problem is to de-duplicate data before building models. The de-duplication can be done by selecting one index admission per patient with equal probability. Another common way is to use generalized estimating equations (GEE). We compare the above two methods with the basic models that simply ignore correlations. We find that neither de-duplication nor GEE is superior to the basic models. Therefore, we decided to focus on the basic models only in this study.

**Table 1:** An example to display the definition of index admissions and readmissions

Patient ID	Encounter ID	Admission date	Discharge date	Elective	Index admission	Readmission	Followed by readmission
1	1	2/25/2011	3/2/2011	N	Y	-	N
1	2	3/25/2011	3/29/2011	Y	Y	N	Y
1	3	4/10/2011	4/13/2011	N	Y	Y	N
1	4	7/22/2012	7/28/2012	N	Y	N	Y
1	5	8/15/2012	8/20/2012	N	N	Y	-

To check the effect of sample size, at each step, we create subsets of data by randomly selecting 1/2, 1/4, 1/8, 1/16 and 1/32 of the original data. Each index admission has equal chance to be included in a subset or not. Then for each subset, we randomly divide data into two equal size subsets: fitting and validating. The stratified k-fold (k ≥ 3) or bootstrap cross-validation can be better choices. But in this paper, we choose to simplify the splitting process and focus on the effect of sample size instead. Predictors are selected based on fitting data only and then are applied to fitting and validating data sets separately. The smallest data set has about 5,000 index admissions and 2,500 for fitting and validating respectively. We make the smallest data set to be 5,000 so that the results can be comparable with the LACE model, which was inferred from 4,812 patients. The random procedures to select various sizes of subsets and to half-split samples for fitting and validating are all independent. AUCs are calculated separately for fitting and validating data and for all four methods. For comparison, we repeat the above process 100 times. The average AUCs are reported and compared.

We choose SAS 9.4 for the STEPWISE logistic regression, the R package “glmnet” for LASSO regression [14] and the R package “mboost” for AdaBoost [12, 13]. In the STEPWISE method, we test multiple thresholds of p-values for entry and removal, which range from 0.001 to 0.1 to cover most of the choices by researchers in practice. In “glmnet”, we report results with the tuning parameter λ = λ_min_ in Table 3 and both λ = λ_min_ and λ = λ_1se_ in Table 4, where λ_min_ is the minimum of the deviance profile and λ_1se_ is the minimum at one standard error of the deviance profile. Normal approximations are used to assess statistical significance when comparing average AUCs from different models.

## Results

As the same in all EMR, data can be missing in several ways that are not necessarily random. Firstly, the value of readmission can be missing due to leaving of patients from a particular hospital, which results in an underestimated readmission rate. We use claim data to identify additional patients who originally registered at Advocate system but were readmitted later at other hospitals. This cannot capture all the readmission events. But it does increase the readmission rate by about 1 %. Secondly, for any independent variable with over 1 % missing records, we create a dummy variable of missing status and fit it in the model to identify possibly biased missing pattern. This is particularly useful for independent variables such as lab results because patients with missing lab values tend to be in normal range and are usually not random. Finally, we assume random missing for the other situations.

The number of observed readmissions in the population is 18,707 (out of 162,466 index admissions), which results in a readmission rate of 11.5 %. The distribution of the dependent and 19 important independent variables is displayed in Table 2. The important variables are defined as those being selected at least 95 % of the time in the three models: STEPWISE, LASSO and AdaBoost when the sample size of fitting data is around 80,000. In addition to the four predictors in LACE model, we identify 4 additional numerical predictors: Number of ER Encounters in Last Year, Number of Inpatient Encounters in Last Year, Braden Score, Polypharmacy, and 9 additional risk factors: Inpatient Encounters in Last Six Months, Not Employed, Discharge to Home Care or SNF, AMA, Albumin Level less than 3.4 g/dL, Leukemia, Malignancy, Renal Failure with Hemodialysis, or History of Alcohol Substance Abuse. The last two variables, Dementia and Trauma, are important predictors, but not risk factors. More detailed discussion can be found in the section of Conclusions.

**Fig. 1:**
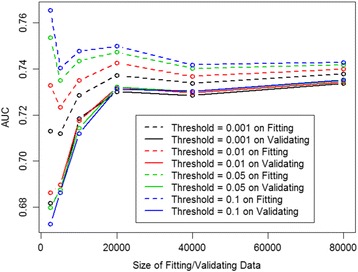
Comparison of AUCs for various choices of threshold (of *p*-value) for entry and removal in the stepwise variable selection procedure

The results from the STEPWISE models are displayed in Fig. 1. We can see that the AUCs on fitting data were higher when the threshold of p-value is larger. However, the influence of this choice on validating data becomes smaller when sample size increases. When sample size increases to 80,000, the AUC with a threshold of 0.1 for validating data is 0.7352, which is slightly, but not statistically significantly, higher than 0.7337 with a threshold of 0.001 (*p* = 0.9). In fact, the AUCs on validating data are all about the same when sample size is at least 20,000. When sample size is less than 5,000, the optimal choice of the threshold is 0.01, which produces the highest AUCs on validating data. In the later comparisons of STEPWISE model to the others, we use results from STEPWISE with threshold being 0.01.

**Table 2:** Characteristics of 162,466 index admissions

	Overall index admissions *n* = 162,466		Index admissions by the value of 30-day all-cause non-elective readmission			
The total degrees of freedom = 24			No (88.5 %) *n* = 143,759		Yes (11.5 %) *n* = 18,707	
	No. or Mean	% or Std.	No. or Mean	% or Std.	No. or Mean	% or Std.
Variables in LACE model						
1. Length of Stay (L)	4.8	4.5	4.6	4.4	5.8	5.3
2. Acuity (A)	123,426	76.0	107,288	74.6	16,138	86.3
3. Charlson Comorbidity Index (C)	2.0	2.4	1.9	2.4	2.8	2.8
4. ER encounters in Last Six Months (E)	33,166	20.4	27,368	19.0	5798	31.0
Additional variables from EMR						
5. Number of ER encounters in Last Year	0.6	2.0	0.5	1.5	1.1	4.1
Index admissions with this value > 0	45,037	27.2	37,532	26.1	7505	40.1
6. Number of Inpatient encounters in Last Year	1.0	1.8	0.8	1.6	2.1	2.9
Index admissions with this value > 0	64,742	39.9	52,998	36.9	11,744	62.8
7. Braden Score	18.6	3.3	18.7	3.2	17.9	3.4
8. Polypharmacy	6.1	5.8	5.9	5.6	7.2	6.5
9. Inpatient encounters in Last 6 Months	51,797	31.9	41,540	28.9	10,257	54.8
10. Employment Status						
Employed	22,007	13.6	20,398	14.2	1609	8.6
Not Employed	83,231	51.2	71,607	49.8	11,624	62.1
Unknown	57,228	35.2	51,754	36.0	5474	29.3
11. Discharge Disposition						
Home/Self care	89,620	55.2	81,757	56.9	7863	42.3
Home Care	26,913	16.6	22,669	15.8	4244	22.7
SNF	29,598	18.2	24,714	17.2	4884	26.1
Rehab	4155	2.6	3780	2.6	375	2.0
LTC, Federal Hospital	9734	6.0	8823	6.1	911	4.9
12. Against Medical Advice (AMA)	2163	1.3	1760	1.2	403	2.2
13. Albumin Level						
< 3.4 g/dL	54,161	33.3	45,778	31.8	8383	44.8
> = 3.4 g/dL (normal range)	34,077	21.0	30,744	21.4	3333	17.8
Unknown	74,228	45.7	67,237	46.8	6991	37.4
14. Leukemia_LMM	3576	2.2	2940	2.1	636	3.4
15. Malignancy	17,384	10.7	14,471	10.1	2913	15.6
16. RF with Hemo	42,844	26.4	35,458	24.7	7386	39.5
17. History of Alcohol Substance Abuse	11,374	7.0	9686	6.7	1688	9.0
18. Dementia	5362	3.3	4688	3.3	674	3.6
19. Trauma	25,241	15.5	22,340	15.5	2901	15.5

The independent variables were those being selected at least 95 % of the time in the three models: STEPWISE, LASSO and AdaBoost when the sample size of fitting data was around 80,000

Next, we compare results from LACE, STEPWISE, LASSO and AdaBoost models. The average AUCs and their 95 % confidence intervals are displayed in Table 3. We exclude results from subsets with size being 10,000 and 40,000 to make the table concise. From this table, we can see that the AUCs based on LACE score were always around 0.65. There are more variations in this number when sample size decreases. There is no significant difference between AUCs from LACE fitting and LACE validating data. The model based on the STEPWISE strategies performs significantly better than the LACE method. When the size of fitting data is less than 20,000, it is obvious that the STEPWISE method tends to over-fit the model because the AUC from validating data set drops significantly when sample size decreases. However, when the size of fitting data is more than 20,000, the AUC from STEPWISE validating data set is stabilized around 0.73, which is 0.28 higher than AUC from LACE validating data. This indicates that making use of additional variables from EMR does significantly increase the performance of predictive models.

**Table 3:** Means and 95 % confidence intervals of AUCs (%) for four readmission risk modelling approaches

	Fitting		Validating	
	Mean	95 % CI	Mean	95 % CI
Fitting/validating data ~ 2,500				
LACE	65.3	(63.0, 67.5)	65.1	(62.8, 67.3)
STEPWISE	73.3	(70.3, 76.3)	68.6	(65.7, 71.6)
LASSO	75.1	(71.0, 79.1)	70.3	(68.0, 72.7)
AdaBoost	82.9	(81.0, 84.8)	67.6	(65.0, 70.3)
Fitting/validating data ~ 5,000				
LACE	64.7	(63.1, 66.3)	64.6	(62.9, 66.2)
STEPWISE	72.3	(70.5, 74.2)	69.0	(67.4, 70.5)
LASSO	73.9	(71.4, 76.3)	70.3	(69.0, 71.7)
AdaBoost	77.9	(76.8, 79.1)	69.4	(67.8, 70.9)
Fitting/validating data ~ 20,000				
LACE	65.2	(64.5, 66.0)	65.3	(64.5, 66.0)
STEPWISE	74.2	(73.4, 75.1)	73.2	(72.4, 74.0)
LASSO	74.9	(74.0, 75.7)	73.5	(72.8, 74.3)
AdaBoost	75.5	(74.7, 76.2)	73.5	(72.8, 74.3)
Fitting/validating data ~ 80,000				
LACE	65.6	(65.2, 65.9)	65.5	(65.2, 65.9)
STEPWISE	74.0	(73.6, 74.4)	73.4	(73.0, 73.9)
LASSO	74.3	(73.9, 74.7)	73.7	(73.3, 74.1)
AdaBoost	74.2	(73.9, 74.6)	73.7	(73.3, 74.1)

In STEPWISE, the threshold for entry and removal is *p* = 0.01. In LASSO, λ = λ_min_

When sample size is less than 20,000, all three variable selection strategies over-fit the model. The AUC of the AdaBoost model is the highest on fitting data, but the lowest on validating data. The STEPWISE logistic model is better and LASSO is the best among the three with the highest AUC on validating data. When sample size is at least 20,000, the prediction performance of STEPWISE, LASSO and AdaBoost models is similar. The AUCs on validating data are all around 0.73-0.74. The STEPWISE method has a slightly lower AUC comparing to LASSO and AdaBoost (73.4 % vs 73.7 %).

In addition, we explore the stability of the selected predictors. For each model, we calculate the mean degrees of freedom (df). We also extract variables that are selected at least 95 % of the time when using the fitting data with size around 80,000. These results are summarized in Table 4. The STEPWISE method with threshold being 0.01 selects the least number of variables on average (df = 67 ± 4). The set of common variables is relatively stable (df = 32). This indicates that although the traditional STEPWISE method can have problems in many situations, it is still reliable to find risk factors for readmissions providing sample size is large enough. The subset of predictors selected by LASSO is not very stable when λ = λ_min_ (df = 190 ± 16). However, when the tuning parameter λ = λ_1se_, the average df can be dramatically reduced (df = 105 ± 10). The average AUC on validating data is still about the same. Therefore, the choice of λ can largely affect the stability of selected predictors in LASSO method and should be carefully explored in practice. The results from AdaBoost are similar to LASSO. The relatively larger pool of predictors in AdaBoost makes it inefficient for the purpose of predictor searching.

**Table 4:** Comparison of the selected set of predictors

	df for models, mean (std)	df for variables selected at least 95 % of the time	Average AUC on validating data (%)
LACE	4 (0)	4	65.54
STEPWISE (*p* = 0.01)	67 (4)	32	73.43
STEPWISE (*p* = 0.1)	112 (11)	35	73.52
LASSO (λ = λ_min_)	190 (16)	72	73.70
LASSO (λ = λ_1sd_)	105 (10)	56	73.61
AdaBoost	157 (5)	63	73.69

Finally, we fit a logistic model using only the 19 variables from Table 1, with a fitting data set of around 80,000. The average AUC on validating data turns out to be 73 %, which is very close to the best results from STEPWISE, LASSO or AdaBoost. This indicates that a large proportion of independent variables might be falsely selected as important predictors when using a single method and a single division of fitting/validating data. It is possible to figure out “true” important predictors using the strategy of repeatedly dividing data into fitting/validating subsets and referring the final model based on summarizing results.

## Discussion

It is interesting to see that the two variables, Dementia and Trauma, are consistently selected for prediction models. In fact, the average coefficients are −0.28 for Dementia and −0.13 for Trauma in the fitted models, which indicates that Dementia and Trauma can decrease readmission risk. However, from Table 2, we observe inconsistent effects: Dementia is more often in encounters with readmissions in 30 days than in encounters without readmissions (3.6 % vs 3.3 %, *p* = 0.013); distribution of Trauma does not differ in encounters with or without readmissions in 30 days (15.5 % vs 15.5 %, *p* = 0.91). A further exploration shows that Dementia patients tend to have lower Braden scores (15.2 vs 18.7, *p* < 0.001) and higher Charlson Comorbidity Index scores (4.9 vs 1.9, *p* < 0.001), while Trauma patients tend to have lower Braden scores (16.6 vs 19.0, *p* < 0.001) and longer length of stays (6.8 vs 4.4, *p* < 0.001). Meanwhile, the variables of Braden Score, Charlson Comorbidity Index and Length of Stay are all very important predictors individually or jointly. Therefore, it was very important to include both variables in the model to improve the prediction performance.

Although our aim here is not to identify an optimal final predictive model, the AUCs on our validating data reaches 0.73-0.74, which is much higher than most published readmission risk predictive models [21]. In fact, according to the extensive literature review on readmission risk predictive models by Kansagara in 2011, the highest reported AUC is 0.83 [6]. However, the dependent variable in Coleman et. al. [6] is complicated care patterns, which is similar but essentially different from the definition of 30-day readmission risk. In addition, patient survey data on functional status and vision were used as predictors. But these variables are usually not ready to use at the time of discharge in medical practice. Of the other models with AUC above 0.7, one study is based on heart failure patients at single center and the others are based on data outside US. In the models tested in large US populations, the AUCs are only 0.55-0.65. Based on a similar data set to ours, the predictive models inferred by the Advocate Cerner Collaboration team in 2013 has an AUC of 0.75 when using a group of predictors with degrees of freedom over 70 [5]. However, the AUC dropped to 0.70 in practice, which was mostly caused by missing data because of a large number of predictors in the model. In addition, the predictive models can become less and less accurate due to hospitals’ aggressive efforts to reduce readmissions in the past two years. With the availability of updated data, refined group of candidate predictors and improved variable selection strategies, we would expect to further enhance our predictive models with possibly less predictors and more reliable performance.

In this paper, we does not focus efforts to deliberately deal with challenges in model fitting such as collinearity within independent variables, nonlinearity between dependent and independent numerical variables, effect of interactions, and etc. However, collinearity within independent variables can be handled relatively well using LASSO or AdaBoost. Nonlinearity can be explored using fractional polynomial functions or discretization of numerical variables. The exploration of interactions is simple in concept, but can have computational difficulties with the increase of sample size and number of independent variables. We will discuss these issues in another paper focusing on the inference of final optimal predictive models.

## Conclusions

We can draw several interesting conclusions from the above comparisons. Firstly, we show that it is very important to make good use of additional variables available from EMR in the readmission risk prediction models to improve accuracy. Secondly, we show that in the STEPWISE selection method, sample size is an important factor in the choice of the threshold of p-value for entry/removal and the performance of predictions. Thirdly, the predictive models from STEPWISE, LASSO and AdaBoost all have similar performance when sample size is large enough. However, the definition of “large enough” varies depending on various choices of models. Our results are based on data from eight Advocate hospitals in the Chicago area. The choices of thresholds and methods for different sample sizes might not be applicable to other populations or predictive models other than readmission risk. However, the strategy to evaluate sufficiency of sample size is applicable to any predictive model on any type of data. When sample size is large enough, all the variable selection methods display very similar prediction power. Some methods perform slightly better when more predictors are considered, which might be less preferred in practice, considering the cumbersome work and lower tolerance for errors. Therefore, it is important to be able to remove falsely selected predictors and focus on important ones only. Our study shows that this can be done by repeatedly dividing data into fitting and validating data sets and retain only variables that are selected most often in the models.
